# Supplementary material for: Beyond single support calls: Efficacy of follow-up callbacks for dementia caregivers using the Alzheimer’s association helpline
Source: PLoS One. 2025 Oct 14;20(10):e0333535. doi: 10.1371/journal.pone.0333535 (PMC12520402; doi:10.1371/journal.pone.0333535)
Supplement: Attachment — Submitted filename: Alzheimers article .pdf [file pone.0333535.s004.pdf]

https://alz-journals.onlinelibrary.wiley.com/doi/epdf/10.1002/alz.082552

PODIUM PRESENTATION
DEMENTIA CARE RESEARCH (RESEARCH PROJECTS; NONPHARMACOLOGICAL)

When One Call Isn't Enough: A Comparison of Alzheimer's Association Helpline Callers

Nancy A Hodgson
University of Pennsylvania, Philadelphia, PA, USA
Correspondence
Nancy A Hodgson, University of Pennsylvania, Philadelphia, PA, USA.
Email: hodgsonn@nursing.upenn.edu

Abstract

Background: Family care partners of persons living with dementia (PLWD) have limited support and resources and experience high levels of stress and burden. Prior research has demonstrated that one call to the Alzheimer's Association's Helpline benefitted 80% of callers following a single call. However, some care partners need more support than a single call.

Method: The aim of this study was to evaluate the efficacy of offering up to two call-backs after an initial call to the Helpline. Study participants were Helpline callers who were asked if they sought additional calls due no progress towards implementing recommended action steps after a single Helpline call. Outcomes were compared at 2, 4and 6 weeks after baseline and included PROMIS measures of self-efficacy.

Result: Of 2504 participants, 79.92% were female, 73% were white, age = 60.31 years (± 12.59), 85.06% bachelor's degree+, 64.42% lived with PLWD. No statistical difference were noted in demographics between single vs. +1 callers. However, +1 callers scored lower on two PROMIS items: upset ($p < 0.0171$) and ability to bounce back ($p < 0.017$). After one call, those with +1 call improved on the total PROMIS ($p < 0.0001$), as well as the following items: negative feelings < 0.001), stress ($p < 0.0009$), upset ($p < 0.0145$), discouragement ($p < 0.006$), and distress ($p < 0.0001$). From the first call to the second call, callers improved on the total score ($p < 0.0065$), upset ($p < 0.0349$) and ability to relax ($p < 0.0213$) items. Following second call, participants improved on the overall survey score ($p < 0.004$), as well as the following items: stress ($p < 0.016$), upset ($p < 0.03$), discouragement ($p < 0.04$), distress ($p < 0.0066$), and ability to relax ($p < 0.02$).

Conclusion: Further research is indicated to understand the support needs of care partners that can benefit from more than one call to a tele support program.

Alzheimer's Dement. 2023;19(Suppl. 19):e082552. © 2023 the Alzheimer's Association.
1 of 1wileyonlinelibrary.com/journal/alz
https://doi.org/10.1002/alz.082552
