# Supplementary material for: Beyond single support calls: Efficacy of follow-up callbacks for dementia caregivers using the Alzheimer’s association helpline
Source: PLoS One. 2025 Oct 14;20(10):e0333535. doi: 10.1371/journal.pone.0333535 (PMC12520402; doi:10.1371/journal.pone.0333535)
Supplement: S1 File — (DOCX) [file pone.0333535.s001.docx]

DATE:	06-Jul-2021	**Institutional Review Board**

TO:	Nancy A Hodgson	3600 Civic Center Blvd., 9th Floor

CC:	Talwar, Sonia

Bujno, Sarah J

RE:

IRB PROTOCOL#: 849227

Philadelphia, PA 19104

Phone: 215-573-2540

(Federalwide Assurance # 00004028)

PROTOCOL TITLE: Alzheimer's Association-Multiple Care Consultation Evaluation Survey

SPONSOR:	ALZHEIMER'S ASSOCIATION REVIEW BOARD: IRB #8

IRB SUBMISSION: NOTICE OF EXEMPTION

Dear Dr. Hodgson,

The above referenced protocol was reviewed by the Institutional Review Board on 01-Jul-2021. It has been determined that the proposal meets eligibility criteria for IRB review exemption authorized by 45 CFR 46.104, category 2.

ONGOING REVIEW:

 The IRB must be kept apprised of any and all changes in the research that may have an impact on the IRB review mechanism needed for a specific proposal. You are required to submit modifications to the IRB if any changes are proposed in the study that might alter the exemption determination, or any applicable HIPAA waiver determination. New procedures that may have an impact on the exemption determination, or HIPAA waiver determination cannot be initiated until Committee approval has been given.

 Consistent with the federal regulations, IRB approval of this protocol will not expire and no continuing reviews will be required for this protocol. The IRB may occasionally contact you to confirm that the trial is still ongoing and that you are adhering the previously stated requirement to submit modifications.

COMMITTEE APPROVALS: You are responsible for assuring and maintaining other relevant committee approvals. This human subjects research protocol should not commence until all relevant committee approvals have been obtained.

If your study is funded by an external agency, please retain this letter as documentation of the IRB’s determination regarding your proposal.

If you have any questions about the information in this letter, please contact the IRB administrative staff. A full listing of staff members and contact information can be found on our website: [http://www.irb.upenn.edu](http://www.irb.upenn.edu/)

***This letter constitutes official University of Pennsylvania IRB correspondence. ***
