# Supplementary material for: Beyond single support calls: Efficacy of follow-up callbacks for dementia caregivers using the Alzheimer’s association helpline
Source: PLoS One. 2025 Oct 14;20(10):e0333535. doi: 10.1371/journal.pone.0333535 (PMC12520402; doi:10.1371/journal.pone.0333535)
Supplement: Attachment — Submitted filename: Response to reviewers.docx [file pone.0333535.s005.docx]

April 11, 2025

Vineet Gupta, MD, FACP, SFHM, CHCQM
Academic Editor
PLOS ONE

Dear Dr. Gupta:

Thank you for your careful review and insightful comments regarding the manuscript:  PONE-D-24-41264- *When One Call Isn’t Enough: A Comparison of Alzheimer’s Association Helpline Callers*. My co-authors and I have revised the manuscript according to the reviewer suggestions for improvement and I have organized my response according to the reviewer’s comments. Thank you for considering this manuscript for publication.

Editor review:

1. In the ethics statement in the Methods, you have specified that verbal consent was obtained. Please provide additional details regarding how this consent was documented and witnessed, and state whether this was approved by the IRB.

*Verbal consent was documented by Helpline agents in their Personify system. This protocol was approved by IRB. This has been amended as suggested in the Methods section.*

2. Please note that funding information should not appear in any section or other areas of your manuscript. We will only publish funding information present in the Funding Statement section of the online submission form. Please remove any funding-related text from the manuscript.

*No funding-related text appears in the manuscript.*

*3.* Please state what role the funders took in the study. (Funding Statement)

*As we now note the funders had no role in study design, data collection and analysis, decision to publish, or preparation of the manuscript. The funder provided support in the form of salaries for coauthors, but did not have any additional role in the study design, data collection and analysis, decision to publish, or preparation of the manuscript. The specific roles of these authors are articulated in the ‘author contributions’ section. We also provide the revised Competing Interests Statement in the Manuscript. We confirm that this commercial affiliation does not alter our adherence to all PLOS ONE policies on sharing data and materials.*

4*. We have uploaded the minimal anonymized data set necessary to replicate study findings.*

*5.* Please include your full ethics statement in the ‘Methods’ section of your manuscript file. In your statement, please include the full name of the IRB or ethics committee who approved or waived your study, as well as whether or not you obtained informed written or verbal consent. If consent was waived for your study, please include this information in your statement as well.

*Thank you. This has been amended.*

Reviewer #1:

- Exclusions are (or seem) intuitive, but it would be nice to know how many were excluded, and/or to “operationalized/define “crisis situation” and “frequent repeat caller” a bit more. If this is left to the discretion of the Helpline agent, then none of that information may be available, but a sentence/phrase with that explanation could be added to the manuscript.
  - *Thank you for this suggestion. The number of participants excluded was not documented by the Alzheimer’s Association Helpline agents. Crisis callers and frequent repeat callers have now been defined.*
- The first paragraph of the discussion could probably be tightened up a bit: “A score of 40 is 1 standard deviation lower than US general population and score of 60 is 1 SD higher than the US general population.” is redundant with the immediately preceding sentence and could just be removed. The subsequent three sentences, “Callers who requested three calls […]” could be condensed something like this: “Compared to that normative data, out caregivers had below-average emotional self-efficacy, especially when requesting multiple consultations (one: 47.1, two: 44.5, three: 39.4).”
  - *We appreciate this comment and have amended this section.*
- “Our first finding indicates that those who later requested additional calls had the lowest levels of self-efficacy in managing emotions at baseline, indicating the highest level of need.” My sense is that “highest level of need” extends beyond that PROMIS can measure. There is more to “need” than emotional self-efficacy, after all. Something like “[…] suggesting an elevated level of need” might better capture the scope of the present work.
  - *Thank you. This has been updated with the suggested language.*
- This paragraph is more of a result than a discussion; the authors should consider moving to another section, or abbreviating or eliminating it, since the information it contains is a retelling of the contents of Table 3:“Five items from the PROMIS Self-Efficacy measure were significant in those who had one or two care consultations. The items “I can handle negative feelings”, “I can find ways to manage stress”, “I can avoid feeling discouraged stress”, and “I can keep emotional distress from interfering with things” were significantly improved (p <0.01) in those who had one care consultation. The item “I can find ways to manage stress” was significantly improved from baseline in those who had two care consultations” (p<0.01).”
  - *Thank you. This has been moved to Results section and briefly summarized in the Discussion section.*
- This study has several limitations. First […]” The authors name only one limitation before proceeding to the next paragraph (“Despite these limitations […]”). …did the authors intend for the later paragraph (“Practice recommendations cannot […]”) to be a second limitation? Another one would be the loss-to-follow-up of almost half the sample (2503 with initial consultation, 1375 of those engaging with the call two weeks later). Another would be that those engaging with subsequent calls may be a self-selected group of people who find benefit from the consultation calls.
  - *Thank you. The Limitations section has been updated with this information*
- “Our second finding suggests that callers benefited from all calls […]” are the authors sure they want to commit to *all*? Every single one? What if some of those 2503 didn’t value the call, and that’s why they were lost to follow-up?
  - *Thank you. This has been amended.*

Reviewer 2:

- First of all, I propose expanding the theoretical background. Although there are few comparable studies, it would be worthwhile to outline in a more exhaustive way the difficult and problematic situations that result from the specificity of dementia (PLWD) in the introduction.
  - *Thank you. We now provide further elaboration of the specific difficulties inherent in dementia caregiving.*
- It would be good to capture the specificity of the functioning of patients and the difficulties experienced by their families. It seems important to me to outline other possible predictors of the effectiveness of using the offered telephone help - those that could be attempted to be identified in future studies.
  - *Thank for your this helpful comment.We have added a discussion of other possible predictors to our concluding statements on future research.*
- I believe that qualitative data, consisting of statements from people using the indicated help, would also contribute a lot. They could better illustrate the problem.
  - *Unfortunately, we do not currently have any recorded/documented qualitative data. We have noted this in the manuscript as a limitation.*
- The methodological part requires some additions. The selection of the sample and presentation of the results do not raise any objections. It seems to me that the PROMIS Self-Efficacy in Managing Emotions Short Form 8A research tool has been described quite poorly and in this form it is difficult to draw conclusions about its reliability.
  - *Thank you. More information has been added about the PROMIS instrument and its reliability.*
- There are possibly not a lot of studies about the non-inferiority of training methods like yours that extend the findings to outcomes for clients eg CG and PLWD, but rater stop at measuring knowledge and confidence etc. This is worth highlighting here somewhere also or in the discussion perhaps.?
  - *Similar to our comment above, we now highlight how this study protocol will evaluate outcomes beyond typical training outcomes of “confidence” and “knowledge”.*
- The authors have correctly identified the limitations of the research. However, I think that the discussion of the results is worth expanding
  - *We appreciate this comment, and we have expanded our discussion of results.*

Thank you. Sincerely,

Nancy A. Hodgson, PhD, RN, FAAN

Claire M. Fagin Leadership Professor in Nursing

Chair and Professor, Department of Biobehavioral Health Sciences
