# Supplementary material for: Beyond single support calls: Efficacy of follow-up callbacks for dementia caregivers using the Alzheimer’s association helpline
Source: PLoS One. 2025 Oct 14;20(10):e0333535. doi: 10.1371/journal.pone.0333535 (PMC12520402; doi:10.1371/journal.pone.0333535)
Supplement: Attachment — Submitted filename: Reviewer response.docx [file pone.0333535.s006.docx]

June 3, 2025

Vineet Gupta, MD, FACP, SFHM, CHCQM

Academic Editor

PLOS ONE

Dear Dr. Gupta:

Thank you for your review and comments regarding the manuscript: PONE-

D-24-41264- When One Call Isn’t Enough: A Comparison of Alzheimer’s Association Helpline

Callers. My co-authors and I have revised the manuscript and title according to the reviewer

suggestions for improvement. Thank you for considering this manuscript for publication.

 Please find our response to editor and reviewer comments below:

1. This work was published as an abstract in December 2023 <https://alz-journals.onlinelibrary.wiley.com/doi/epdf/10.1002/alz.082552> that needs to be duly acknowledged and cited as feasible. Even though it was your own work and presented in part- missed that important piece may be misconstrued as omission. File is attached for your reference.

*Thank you for your attention to this. We presented early data from this study in 2023. We did not use the full dataset and these were preliminary findings presented as an abstract.*

2. Also, in the abstract 2504 participants were included that is 2503 in this manuscript- any reason for this discrepancy?

*We appreciate your attention to this detail. The work presented in the abstract was preliminary and did not include the final dataset. For the final analysis, we identified rules for duplicates and data missingness and this changed the number of included participants in this final analysis.*

3. Abstract can include more information in methods section (some mention of statistical analyses or anything relevant in methodology) and results (Additional relevant information).

*Thank you for this feedback. We have updated the abstract with additional information in these sections.*

1. Please review your reference list to ensure that it is complete and correct.

*We have reviewed the reference list. We removed a citation because it was no longer cited in the text.*

2. First of all, I would like to note that the author's article with the same title has been published in the meantime in the journal Alzheimer's & Dementia 19(S19),December 2023 DOI: 10.1002/alz.082552. I have not had the opportunity to compare the content (from the abstract I conclude that they may concern exactly the same study), but the fact that a text with the same title exists is the basis for changing the title. Change title (minor change)

*Thank you for this recommendation. We have now changed the title to “Beyond Single Support Calls: Efficacy of Follow-Up Callbacks for Dementia Caregivers Using the Alzheimer's Association Helpline”.*

Sincerely,

Nancy A. Hodgson, PhD, RN, FAAN
